# Diazotrophic Bacteria Is an Alternative Strategy for Increasing Grain Biofortification, Yield and Zinc Use Efficiency of Maize

**DOI:** 10.3390/plants11091125

**Published:** 2022-04-21

**Authors:** Arshad Jalal, Carlos Eduardo da Silva Oliveira, Henrique Benetasse Fernandes, Fernando Shintate Galindo, Edson Cabral da Silva, Guilherme Carlos Fernandes, Thiago Assis Rodrigues Nogueira, Pedro Henrique Gomes de Carvalho, Vinícius Rodrigues Balbino, Bruno Horschut de Lima, Marcelo Carvalho Minhoto Teixeira Filho

**Affiliations:** 1 Department of Plant Protection, São Paulo State University (UNESP), Rural Engineering and Soils (DEFERS), Ilha Solteira 15385-000, SP, Brazil; arshad.jalal@unesp.br (A.J.); ces.oliveira@unesp.br (C.E.d.S.O.); henrique.b.fernandes@unesp.br (H.B.F.); guilherme.carlos.fernandes@gmail.com (G.C.F.); tar.nogueira@unesp.br (T.A.R.N.); pedro.goomes04@gmail.com (P.H.G.d.C.); viniciusrbalbinoega@gmail.com (V.R.B.); bruno.horschut@unesp.br (B.H.d.L.); 2 Center for Nuclear Energy in Agriculture (CENA), University of São Paulo (USP), Piracicaba 13416-000, SP, Brazil; fs.galindo@yahoo.com.br; 3 Rio Verde Campus, Goiano Federal Institute, Rio Verde 75901-970, GO, Brazil; edsoncabralsilva@gmail.com

**Keywords:** *Zea mays* L., agronomic biofortification, zinc uptake, productivity, zinc efficiencies, zinc nutrition, inoculation

## Abstract

Biofortification of cereal crops with zinc and diazotrophic bacteria is a sustainable solution to nutrient deficiency and hidden hunger. The inoculation of staple grain crops such as maize is increased with reducing productivity losses while improving nutrition and use efficiency under climatic extremes and weathered soils of tropical savannah. Therefore, objectives of our study were to evaluate the influence of seed inoculation with diazotrophic bacteria (No inoculation–Control, *Azospirillum brasilense*, *Bacillus subtilis*, and *Pseudomonas fluorescens*) together with residual effect of soil Zn (absence and presence) on growth, yield, Zn nutrition, Zn use efficiencies, and intake of maize in 2019 and 2020 cropping seasons. The inoculation of *B. subtilis* increased hundred grain mass and yield (14.5 and 17%), while *P. fluorescens* under residual Zn fertilization has improved shoot and grain Zn concentration in shoot (29.5 and 30.5%). and grain (25.5 and 26.2%), while improving Zn accumulation in shoot (33.8 and 35%) and grain (37.2 and 42%) of maize. The estimated Zn intake in maize was also increased with *A. brasilense* inoculation and residual Zn application. The Zn use efficiencies including Zn use efficiency, agro-physiological, and utilization efficiency was increased with *B. subtilis*, while applied Zn recovery was increased with *A. brasilense* inoculations under residual Zn fertilization. Zinc use efficiency was increased by 93.3 and 397% with inoculation of *B. subtilis* regardless of Zn application. Therefore, inoculation with *B. subtilis* and *P. fluorescens* along residual Zn fertilization is considered the most effective and sustainable strategy for agronomic biofortification of maize under harsh tropical conditions of Brazil.

## 1. Introduction

Maize (*Zea mays* L.) is a crop of social and economic importance, and feeds more than 65% of the global population with a sustainable intake of proteins and calories [1]. It is a versatile cereal cultivated in diversified environments due to its changing food habits and increasing consumption by non-vegetarians [2]. Brazil is the 3rd largest producer of maize around the world with a production of 95 million tons from 17.5 million hectares [3], but still low in average yield production as compared to American and European regions [4]. Maize is inherently poor in minerals’ concentration which is usually plagued by a widespread zinc (Zn) deficiency in tropical regions and ultimately confronts plants’ nutrient acquisition, productivity, and food quality as well as human nutrition and health [5].

Zinc deficiency is a global threat, affecting one third of agricultural soils, and leading to poor production and nutritional quality of cereal crops [6]. Zinc soil deficiency is mainly caused by its abundant soil silicate, oxide, phosphate, and carbonates in soil as well as extensive farming and chemical fertilization, and inadequate irrigation [7]. In addition, Zn is the most transitional nutrient for plant physiological processes, protein synthesis, energy production, genes expression, photosynthesis, and enzymatic activities, as well as pollen fertility, and hormonal and carbohydrate metabolism while discouraging pathogen infestation in cereal crops [8]. Besides this, cereals based on low Zn nutritional security are mainly contributing to human Zn deficiency, and have become the challenge of the day, especially in developing countries [9] and tropical soils [10]. This has introduced the requirement of ultra-nourished strategy such as agronomic biofortification to alleviate malnutrition with effective improvement of nutrition and dietary consumption for the targeted population [11]. However, biofortification of crops with single nutrient in soil application may not be enough for better growth, nutrition, and productivity under harsh tropical conditions. Thus, microbes-mediated biofortification of field crops is an ecofriendly and sustainable strategy to better understand transport of nutrients to grains with greater productivity and nutrient use efficiency [10].

Several plant growth promoting bacteria (PGPBs) are being involved in stimulation of different direct (nutrient acquisition and growth stimulation) and indirect mechanisms (stress diminution and bio-control resistance) that can improve nutrient cycling, maintaining homeostasis, and decomposition of organic material with greater crop production under a sustainable and ecofriendly environment [12]. These rhizosphere bacteria unlocked Zn in the soil by establishing association with plant roots for better accessibility to support plant growth and production [7]. These microbes of synthesis chelating compound in the rhizosphere of roots where they form complexes with Zn and increase its availability and consequently, biofortification through production of siderophores, indole acetic acid (IAA), gibberellins and cytokinins, and reducing phytic acid [12,13]. Several genera of beneficial bacteria such as *Rhizobium*, *Pseudomonas*, *Azospirillum*, and *Bacillus* are being quoted as Zn solubilizer that facilitate translocation of Zn from soil to different plant tissues, promoting productivity and enriching grains, thus supporting ecofriendly agronomic biofortification [7,14,15]. Inclusion of Zn solubilizing bacteria is the most competent, feasible, and least expensive strategy for Zn biofortification of edible grains (especially maize) with admirable results on sustainable agriculture [16].

Maize is currently the largest cereal source in the world, therefore, it is required to determine sources and dissemination of Zn uptake in maize grains for better understanding of its performance on global Zn cycling. The literature is lacking with Zn biofortification of maize under the interaction of diazotrophic bacteria and soil applied Zn in tropical Savannah. There is also a research gap on the association of diazotrophic bacteria and residual Zn fertilization on Zn nutrition, Zn use efficiency (ZnUE), and yield of maize crop. The integrated use of diazotrophic bacteria and chemical fertilizer is an emerging alternative in the agricultural world. Therefore, it was hypothesized that inoculations of diazotrophic bacteria may have synergetic relation with residual Zn application on plant and grain concentrations, growth, yield, ZnUE, and daily intake of biofortified maize grains in tropical Savannah of Brazil. Therefore, the specific objectives of this study were to better evaluate the performing diazotrophic inoculant in the presence and absence of residual soil Zn fertilization on maize growth, leaf, and grain Zn concentration, and accumulation, yield, and Zn use efficiencies in two consecutive growing seasons.

## 2. Results

### 2.1. Plant Height, Dry Matter and Grain Yield

The insertion of first productive cob, plant height, shoot dry matter, and grain yield of maize were significantly increased with diazotrophic bacterial inoculation in residual Zn applied treatments as compared to without Zn residual treatments (Table 1). The residual Zn applied treatments produced taller plants with elevated insertion of productive cob (first cob insertion) as compared to control. The plant height was increased by 1.9 and 2.2% in 2019–2020 and 2020–2021 cropping seasons, respectively. Seed inoculation with *A. brasilense* increased plant height by 4.5%, while height of productive cob was increased by 5.7 and 6.4% with seed inoculation of *P. fluorescens* as compared to control in both cropping seasons, respectively. 

**Figure 1:**
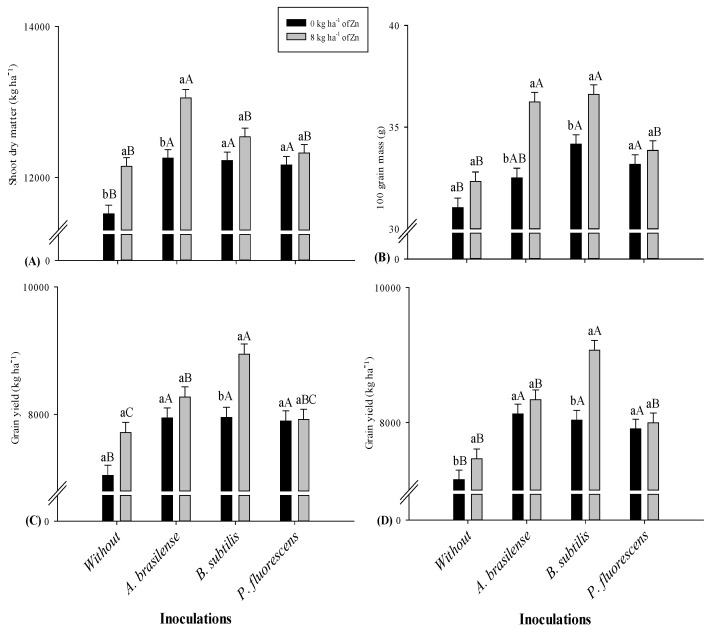
Maize shoot dry matter (**A**) and 100 grains mass (**B**) in 2020–2021 respectively, and grain yield in 2019–2020 (**C**) and 2020–2021 (**D**) as function of residual Zn doses and diazotrophic bacteria. Without = control (no inoculation). The uppercase letters are used for inoculation interactions within each level of soil applied residual Zn whereas lowercase letters are used for the interactions of Zn doses (presence and absence) within each inoculation treatment. The identical alphabetic letters do not differ from each other by Tukey test (*p* < 0.05) for Zn doses and inoculations in 2019–2020 and 2020–2021. Error bars indicate standard error of the mean (*n* = 4 replications).

Shoot dry matter was significantly greater with residual Zn application and bacterial inoculation in 2019–2020 and 2020–2021 cropping seasons (Table 1). Residual Zn applied treatments were observed with greater shoot dry matter (3.7 and 3.9%) as compared to control treatments in both cropping seasons. The treatments with inoculation of *A. brasilense* were noted with greater dry matter (5.8 and 6.9%), which were statistically at per with treatments of *B. subtilis* and *P. fluorescens* in both studied cropping seasons. The interaction of residual Zn doses and bacterial inoculations for shoot dry matter was significant only in the second cropping season (Figure 1A). 

Hundred grains mass and grain yield of maize were significantly increased with residual Zn application and diastrophic bacteria inoculations in 2019–2020 and 2020–2021 cropping seasons (Table 1). The single effect of residual Zn (8 kg ha^−1^) increased mass of 100 grains by 5.9 and 6.4% in relation to control. The inoculation with *A. brasilense* produced heavy 100 grains (9.5%), which was statistically similar to treatments of *B. subtilis* and *P. fluorescens* in 2019–2020 maize harvest in comparison to control. The treatments inoculated with *B. subtilis* increased 100 grains mass by 11.7% in 2020–2021 maize harvest which was statistically at per with treatments of *A. brasilense* as compared to control. The interaction for 100 grains mass was significant only in second harvest (Figure 1B). In addition, residual Zn applied treatments increased grain yield by 6.6 and 5.3% while inoculation with *B. subtilis* increased by 14.5 and 17.1% in 2019–2020 and 2020–2021 cropping seasons as compared to control (Table 1). The interactions for grain yield were significant in both cropping seasons (Figure 1C,D). The seed inoculation with *B. subtilis* was observed with greater grain yield under residual Zn application while *A. brasilense* treatments were noted with greater grain yield in the treatments without Zn fertilization (control) in both maize harvests. The treatments with *P. fluorescens* and the control were observed with lower grain yield regardless of the Zn fertilization in both years of crop harvest (Figure 1C,D).

### 2.2. Zinc Concentration in Leaf, Shoot, and Grains

**Figure 2:**
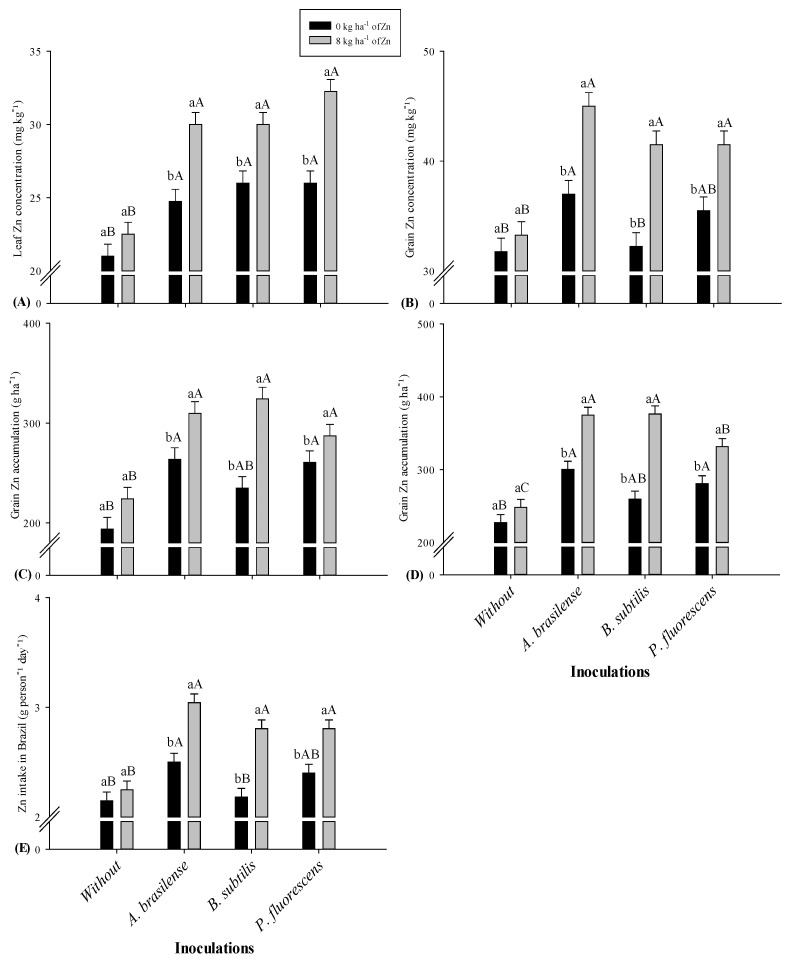
Leaf zinc concentration (**A**) and grain zinc concentration (**B**) in 2020–2021, respectively, and grain zinc accumulation in 2019–2020 (**C**) and 2020-2021 (**D**), and estimated daily zinc intake in Brazil in 2020−2021 cropping maize season (**E**) as function of residual Zn doses and diazotrophic bacteria. Without = control (no inoculation). The uppercase letters are used for inoculation interactions within each level of soil applied residual Zn whereas lowercase letters are used for the interactions of Zn doses (presence and absence) within each inoculation treatment. The identical alphabetic letters do not differ from each other by Tukey test (*p* < 0.05) for Zn doses and inoculations in 2019–2020 and 2020–2021. Error bars indicate standard error of the mean (*n* = 4 replications).

Residual Zn application and bacteria inoculation significantly increased leaf, shoot, and grain Zn concentrations of maize in 2019–2020 and 2020–2021 cropping seasons (Table 2). Leaf Zn concentration was increased by 15.2 and 17.6% under soil applied Zn doses in relation to control in the first and second cropping seasons, respectively. Inoculation with *P. fluorescens* was observed with higher leaf Zn concentration (23.8 and 34.1%), which were statistically at per with the treatments inoculated with *B. subtilis* and *A. brasilense* in both cropping seasons. The interaction of residual Zn and bacterial inoculations for leaf Zn concentration was significant only in the 2020–2021 cropping season (Figure 2A). The inoculations of all studied bacteria increased leaf Zn concentration in the presence of residual Zn fertilization. However, inoculation with *P. fluorescens* was observed for higher leaf Zn concentration in the presence of residual Zn fertilization while *A. brasilense* with lower in the absence of Zn fertilization (Figure 2A). 

Maize shoot Zn concentration was increased by 15.2 and 15.7% as a function of residual Zn fertilization in 2019–2020 and 2020–2021 harvest as compared without Zn fertilization treatments (Table 2). The inoculation with *P. fluorescens* increased shoot Zn concentration by 29.5 and 30.5% in the first and second harvest as compared to control, which were statistically similar to the treatments inoculated with *B. subtilis* and *A. brasilense* (Table 2).

Grain Zn concentration in 2019–2020 and 2020–2021 maize harvest were significantly increased by 12.7 and 18.2% under 8 kg ha^−1^ soil residual Zn fertilization while inoculation with *A. brasilense* increased grain Zn concentration by 25.5 and 26.2% as compared to control (Table 2). The interaction for grain Zn concentration was significant only the second cropping season (Figure 2B). The inoculation with *A. brasilense* was noted for higher grain Zn concentration regardless of the residual Zn fertilization, while treatments with *B. subtilis* were observed with low grain Zn concentration in the absence of Zn fertilization (Figure 2B). 

### 2.3. Zinc Shoot and Grain Accumulation, Partitioning, and Intake in Maize

Zinc accumulation in shoot and grain, partitioning index, and estimated Zn intake in maize consumption were significantly influenced by residual Zn doses and diazotrophic bacteria inoculations in maize cropping seasons of 2019–2020 and 2020–2021 (Table 3). Shoot Zn accumulation in maize was improved by 19.2 and 20.5% with residual Zn fertilization in first and second maize harvest, respectively. Treatments with inoculation of *P. fluorescens* were observed with better accumulation of Zn in shoot (33.8 and 35%) in relation to control, which were statistically similar to the values obtained in treatments with *A. brasilense* in both maize harvest seasons (Table 3).

Residual Zn fertilization in 2019–2020 and 2020–2021 maize cropping season improved grain Zn accumulation by 20.2 and 24.6% (Table 3). Inoculation with *A. brasilense* was noted with higher grain Zn accumulation (37 and 42%) in first and second maize cropping seasons, which was statistically at per with inoculated treatments of *B. subtilis* and *P. fluorescens* in 2019–20202 maize harvest, and with *B. subtilis* in 2020–2021 maize harvest. The interactions of the study factors for grain Zn accumulation were significant in both cropping seasons (Figure 2C,D). Inoculation with *A. brasilense* and *B. subtilis* tended to perform better for grain Zn accumulation under residual Zn fertilization while *B. subtilis* in the absence of residual Zn fertilization was observed with low grain Zn accumulation in both crop harvests (Figure 2C,D).

Zinc partitioning index was not significantly influenced by residual Zn fertilization and bacteria inoculation (Table 3). The treatments with residual Zn fertilization and bacteria inoculations were not statistically different, however, inoculation with *P. fluorescens* performed better in partitioning Zn to grains from low Zn in soil, which was statistically similar to non-inoculated treatments. 

The estimated daily Zn intake in maize consumption in Brazil was significantly increased with residual Zn fertilization and diazotrophic bacteria inoculation in 2019–2020 and 2020–2021 maize harvest seasons (Table 3). The residual Zn fertilization increased daily Zn intake by 14.3 and 17.4% in the first and second cropping seasons as compared to control.

The treatments with inoculation of *A. brasilense* tended to increase Zn intake by 26.3 and 22.7% in 2019–2020 and 2020–2021 maize harvest, which were statistically similar to the treatments with *B. subtilis* and *P. fluorescens* in first season, and with *P. fluorescens* in second maize copping season. The interaction for daily Zn intake was significant only in the second cropping season (Figure 2E). Inoculation with *A. brasilense* tended to increase Zn intake in daily maize consumption regardless of the Zn fertilization, while *B. subtilis* was observed with lower daily Zn intake in the absence of residual Zn fertilization (Figure 2E).

### 2.4. Zinc Efficiencies

Zinc efficiencies such as Zn use efficiency, agro-physiological efficiency, utilization efficiency, and applied Zn recovery were positively increased by diazotrophic bacteria inoculations in residual Zn fertilization (Table 4). Zinc use efficiency (ZnUE) of maize was increased with inoculation of *B. subtilis* in the treatments applied with residual Zn fertilizations in the first and second maize cropping seasons (Table 4). The lower ZnUE was observed in the treatments without inoculations.

Agro-physiological efficiency (APE) was statistically not significant in 2019–2020 maize cropping season. Interestingly, APE of maize was significantly increased with inoculation of *B. subtilis* under residual Zn fertilization in 2020–2021 cropping seasons as compared to control (Table 4). The highest APE was observed in the treatments of *B. subtilis* while the lowest was recorded in treatments of *P. fluorescens* inoculation (Table 4).

Zinc utilization efficiency (UE) was increased by 77.4 and 190.8% with seed inoculation of *B. subtilis* in residual Zn fertilization in relation to non-inoculated treatments, which were statistically similar to the treatments of *A. brasilense* in the first and second maize cropping seasons, respectively (Table 4). The highest Zn utilization efficiency was observed with *B. subtilis* while the lowest was noted in control treatments (Table 4).

Inoculation with *A. brasilense* under residual Zn fertilization performed better in recovery of applied Zn fertilization in 2019–2020 and 2020–2021 maize cropping seasons. Applied Zn recovery was increased by 191.6 and 213.3% in the treatments with residual Zn fertilization and *A. brasilense* inoculation, which were statistically similar to the treatments inoculated with *B. subtilis* and *P. fluorescens* in both crop harvests (Table 4). The lowest applied Zn recovery was observed in control (without inoculation) treatments. 

### 2.5. Pearson’s Correlation among Evaluated Attributes of Maize

**Figure 3:**
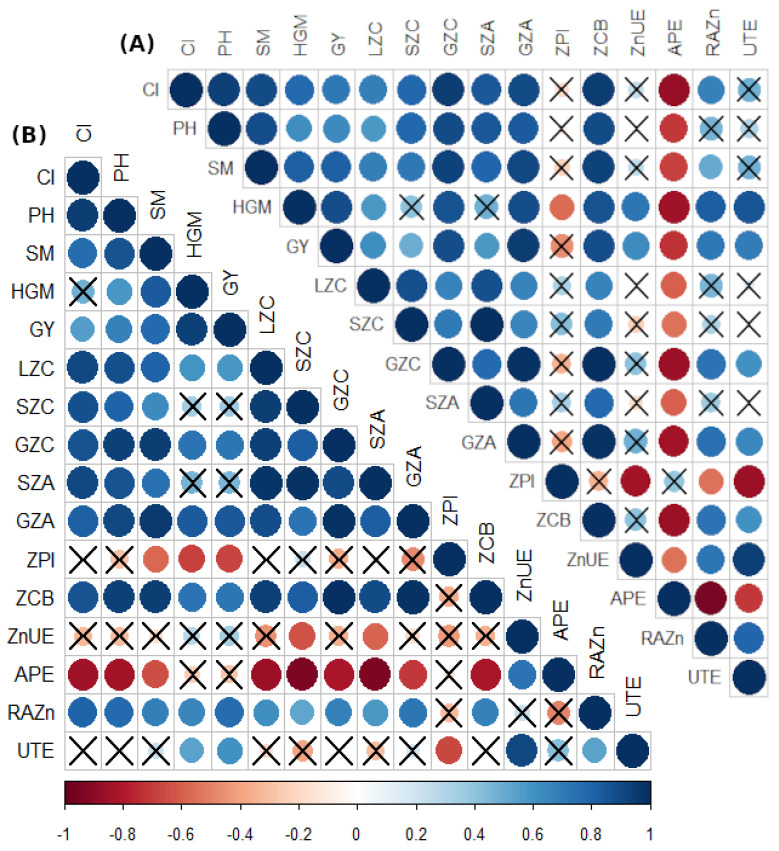
Heat-map color scale indicating Pearson’s correlation among evaluated parameters of maize plants in response to residual soil Zn applications and diazotrophic bacteria inoculations in 2019–2020 (**A**) and 2020–2021 (**B**) cropping seasons. X = indicates a non-significant relationship (*p* ≤ 0.05). Abbreviations: CI = Insertion of first productive cob, PH = plant height, SM = shoot dry matter, GY = grain yield, LZC = Leaf Zn concentration, SZC = Shoot Zn concentration, GZC = Grain Zn concentration, SZA = Shoot Zn accumulation, GZA = Grain Zn accumulation, ZPI = Zn partitioning index, ZCB = Zn intake in Brazil, ZnUE = Zn use efficiency, APE = Agro-physiological efficiency, RAZn = Applied Zn recovery, and UTE = Utilization efficiency.

There were overall positive and significant correlations among zinc concentrations in maize plants (leaf, shoot, and grains) and insertion of first productive cob, plant height, shoot dry matter shoot, and grain Zn accumulation, and negative correlation with agro-physiological efficiency, while non-significant correlations with zinc partitioning index, zinc use efficiency, applied zinc recovery, and utilization efficiency (Figure 3A). A positive correlation was observed between leaf, shoot, and grain concentration, and shoot and grain Zn accumulation, daily Zn intake, applied Zn recovery, plant height, shoot dry matter, insertion of productive cob, and grain yield. A negative correlation was noted between Zn partitioning index and plant height, shoot dry matter, insertion of first productive cob, leaf, shoot, and grain concentration, and shoot and grain Zn accumulation, and daily Zn intake. A non-significant correlation was noted between zinc utilization efficiency and Zn partitioning index, zinc use efficiency, and 100 grains mass (Figure 3B).

## 3. Discussion

Agronomic biofortification has been recognized as the most feasible and effective mechanism for correcting zinc (Zn) deficiency in soil and plant along with better quality yield to improve human health. Single Zn fertilization could not facilitate Zn soil, plant, human, and environment at the same time, especially in tropical regions [10,17]. Therefore, integrated use of bio- and mineral fertilizers is an emerging strategy that can mediate nutrient acquisition for soil-plant-human health to facilitate millions in the population in a sustainable and ecofriendly manner. Diazotrophic bacteria are colonializing root rhizosphere to soluble mineral nutrients, stimulating plant growth with greater yield as well as improving acquisition of nutrient to edible grains [18]. The positive correlation between zinc concentrations in maize plants (leaf, shoot, and grains) and insertion of first productive cob, plant height, shoot dry matter, grain yield, and shoot and grain Zn accumulation validated the hypothesis of the current study (Figure 3). 

Zinc is an essential element of cell development and multiplication as well as pollen fertility for better plant establishment, growth, and reproduction, where its deficiency can plague growth and yield [19,20]. However, the integrated application of diazotrophic bacteria such as Zn solubilizing bacteria and Zn fertilization is one of the best alternative and sustainable strategies to improve Zn nutrition with greater growth and productivity [10,16]. Therefore, the current results verified that residual Zn fertilization and inoculation of *A. brasilense* and *P. fluorescens* has increased plant height, height of insertion of first productive cob, dry matter, and hundred grains and grain yield of maize (Table 1; Figure 1). Several previous studies reported that Zn solubilizing bacteria can rapidly colonialize in root rhizosphere, where they could increase Zn solubilization by producing siderophores, chelators, and several plant growth hormones such as indole acetic acid (IAA), gibberellins, and cytokinins that are immensely linked to better plant health, growth, and production [16,21]. Zinc solubilizing bacteria such *A. brasilense* [17] and *P. fluorescens* [22], together with Zn fertilization, are being reported with greater growth and yield of cereal crops. 

Maize grains are inherently low in Zn concentration which can further hinder nutrient acquisition and yield [5] in Zn-deficient soils. The adequate Zn concentration in maize leaf is ranging from 15–50 mg kg^−1^, below this is considered adequate [23]. Plants and microbes interactions in root rhizosphere stimulate nutrient cycling by solubilization, mineralization, carboxylation, and hormones synthesis [24,25], which could empower Zn concentration and uptake in cereals to support biofortification [22]. Thus, our results verified that residual Zn fertilization and inoculation with *P. fluorescens* and *A. brasilense* has increased concentration in leaf, shoot, and grains (Table 3; Figure 2A,B), and Zn accumulation in shoot and grains of maize (Table 4; Figure 2C,D). The reason might be the presence of microbes in root rhizosphere which could interact with applied inoculants to stimulate transportation of nutrients (especially Zn) to leaf, shoot, and grains by modifying root architecture, secreting phenolic acids, and reducing phytic acid supply to grains [26]. Several other studies exhibited that different strains of *Azospirillum*, *Bacillus*, and *Pseudomonas* sp. promote availability and solubilization of nutrients by synthesis of different plant hormones and enzymes, as well as biological nitrogen fixation [27,28]. Abadi et al. [29] exhibited that inoculants of *Pseudomonas* sp. could alleviate Zn deficiency by increasing root branching and proliferation for greater Zn accumulation and better plant health under harsh environmental circumstances. 

Zinc is an important nutrient of several biological and anabolic processes of humans, while its deficiency could lead to several disorders and hidden hunger [30]. Zinc is an indispensable element for plants and humans to perform their functions and increase productivity [31]. In addition, most of the population are consuming cereals to meet their daily food requirements and therefore, an urgent-based approach such as microbes-mediated Zn biofortification of staple crops can be the most authentic strategy to increase Zn concentration in edible crops under Zn-deficient soils [22]. In this context, the current research indicated that residual Zn fertilization and bacterial inoculation has increased estimated daily Zn intake in Brazil while Zn partitioning was not statistically different (Table 3; Figure 2E). The fact may be the activation of different mechanisms such as acidification, exchange reactions, chelation, and release of organic matter by soil microbes to solubilize nutrients (especially Zn) for better uptake in edible parts [32]. The strains of *Azospirillum*, *Pseudomonas*, and *Bacillus* sp. are being observed with increasing daily intake and partitioning of Zn from soil to grains of different cereal crops [22,33]. It has also been described that inoculation of wheat Zn solubilizing bacteria could increase root volume, diameter, length, and surface area that has ultimately increased Zn uptake by two folds in edible grains [34]. These microbes had increased bioavailability and transportation of Zn to edible grains by reducing phytic acids, which could substantially increase human consumption in a more green and sustainable manner [14,22].

Zinc efficiencies such as Zn use, agro-physiological, and utilization efficiency, as well as applied Zn recovery have differently responded to inoculation and residual Zn fertilization (Table 4). These efficiencies are derived from shoot and grain Zn concentration in Zn-deficient soils, where only Zn fertilization is a fraction of Zn use efficiency while increasing fertilizer dose could decrease Zn efficiency [35]. Most of the studied efficiencies were increased with inoculation of *B. subtilis* under residual Zn fertilization (Table 4). This increase might be due to greater growth, yield, and Zn uptake in the current experiment (Table 1, Table 2 and Table 3). Roots and soil Zn interaction are severally decreased due to low soil moisture and organic matter that can limit Zn absorption, however, greater root dry matter can scavenge and intercept nutrients into plants [36], which is the main access point to increase nutrient uptake and assimilation with higher Zn use efficiency. Nutrient use efficiency is not only dependent on nutrient uptake by plants from soil but is also dependent on growth stage, internal transportation, recycling, and mobilization. Several microbes have capability to increase use efficiency of nutrients by solubilization, where application of minerals and inoculation have promising roles to increase nutrient use efficiency in nutrient-deficient soils [37]. Several studies reported that seed inoculation with strains of *Bacillus*, *Pseudomonas*, and *Azospirillum* enhanced Zn translocation to grains with higher Zn use, agro-physiological, and utilization efficiency, and applied Zn recovery in cereal crops [17,22]. 

## 4. Materials and Methods

### 4.1. Experimental Site and Climate Description

**Figure 4:**
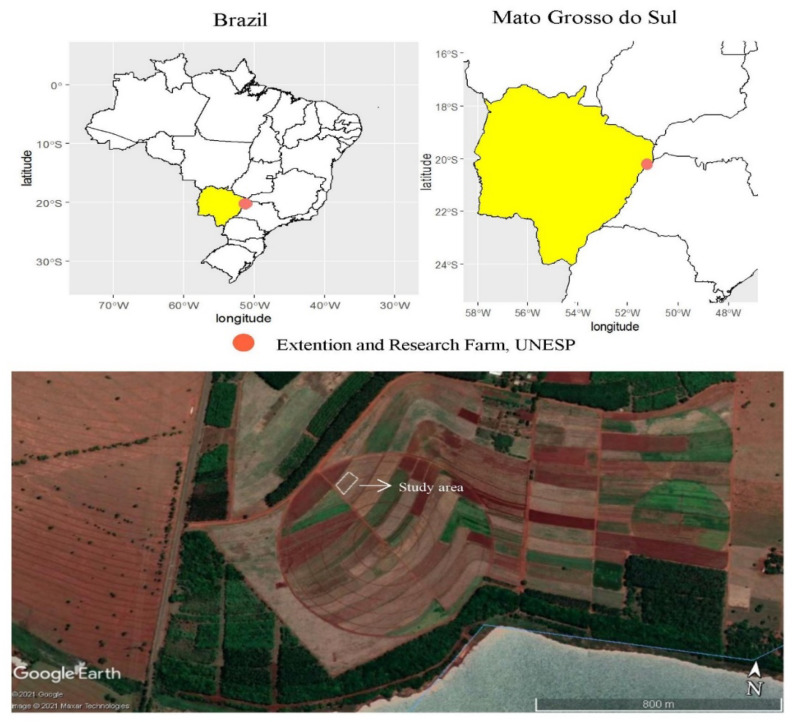
Location of the experimental area at Extension and Research Farm, UNESP—Ilha Solteira Campus, at Selvíria, state of Mato Grosso do Sul, Brazil (20°22′ S, 51°22′ W, altitude of 335 m) in 2019–2020 and 2020–2021 crop seasons. The map was created by using geographic information system (QGIS) software and the Google Earth program. The QGIS Development Team (2021). Open Source Geospatial Foundation project. http://qgis.osgeo.org, accessed on 27 February 2022. Projection System WGS 84/UTM 200DC (EPSG: 4326). This image was taken from the Google Earth program, Google Company (2021). From map data: Google, Maxar Technologies.

**Figure 5:**
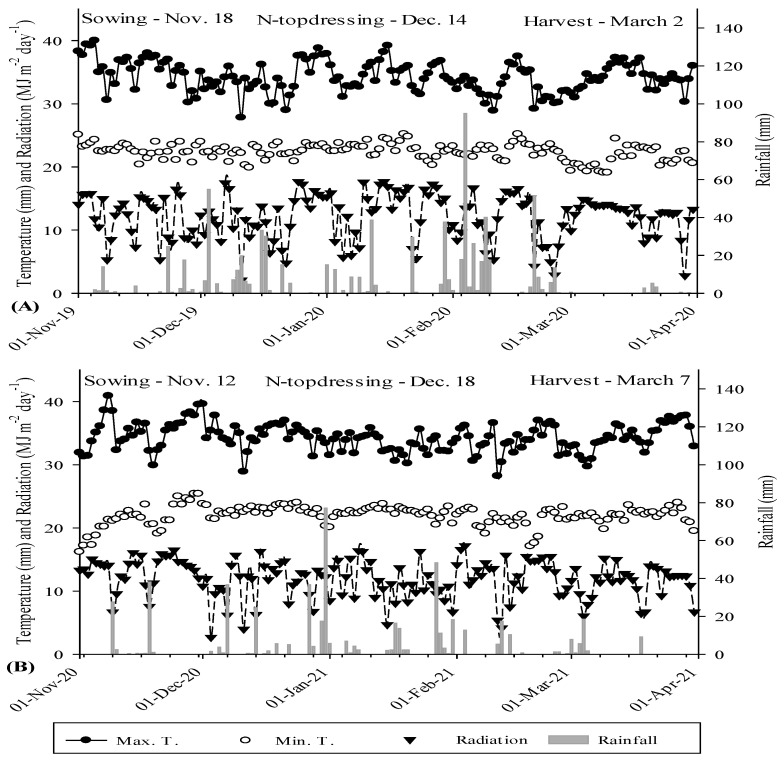
Rainfall, maximum, and minimum temperatures, and light radiation were acquired from the weather station of Extension and Research Farm of School of Engineering—UNESP during maize cultivation period from November to March 2019–2020 (**A**) and 2020–2021 (**B**).

Two years of maize (*Zea mays* L.) field experiments were conducted at Research and Extension Farm of School of Engineering, São Paulo State University (UNESP) in Selvíria, state of Mato Grosso do Sul, Brazil, at geographical coordinates of 20°22′ S latitude, 51°22′ W longitude, and an altitude of 335 m above sea level (Figure 4). The site has been cultivated with cereals-legumes cropping system for more than 28 years, previously cultivated with wheat, wherein the last 13 years were under a no-tillage system. The soil of the experimental site is classified as Rhodic Haplustox [38] and Red Latosol Dystrophic with a clayey texture [39]. The climate of the site is classified as humid tropical of Aw type, rainy in summer, and dry in winter according to Koppen climate classification [40]. The rainfall, minimum, maximum, and average temperatures, and air humidity of maize cultivation period is summarized in Figure 5. 

### 4.2. Soil Analysis

Twenty random soil samples were collected before experiment installation with cup auger from 0.00–0.20 m soil layer. The samples were mixed to make a homogeneous/composite sample for the determination of chemical [41] and granulometric characterization such as clay = 433, sand = 470, and silt = 90 g kg^−1^ following the methodology of Teixeira et al. [42]. The chemical and physical characterization of the site are summarized in Table 5.

### 4.3. Experimental Design and Treatments

The experiments were designed in randomized complete blocks in 4 × 2 factorial scheme with four replications. The treatments consisted of four seed inoculations with diazotrophic bacteria (no inoculation, *Azospirillum brasilense*, *Bacillus subtilis*, and *Pseudomonas fluorescens*) and two residual zinc (Zn) applications (without 0 kg ha^−1^ of Zn and with 8 kg ha^−1^ of Zn), applied from zinc sulphate (21% Zn and 10% S). 

The inoculation of *A. brasilense* strains Ab-V5 and Ab-V6 (Ab-V5 = CNPSo 2083 and Ab-V6 = CNPSo 2084 with guarantee of 2 × 10^8^ CFU mL^−1^) was conducted at a dose of 200 mL ha^−1^ (liquid inoculant) added in a small quantity of water to uniformly mix in around 24 kg of maize seeds sown ha^−1^. The *B. subtilis* (strain CCTB04 with guarantee of 1 × 10^8^ CFU mL^−1^) and *P. fluorescens* (strain CCTB03 with guarantee of 2 × 10^8^ CFU mL^−1^) were performed at a dose of 150 mL ha^-1^ (liquid) according to the recommendation of an inoculants providing company (Total Biotechnology®), Curitiba, Brazil. The inoculations were performed an hour before plantation of the crop, followed in both cropping seasons. 

Zinc fertilization (0 and 8 kg ha^−1^) was performed only in 2019 and 2020 (May to September both years) of wheat cropping seasons. Zinc sulphate was manually applied to soil surface between rows of wheat at V1/V2 stage (one to two completely unfolded leaves) and followed by 14 mm irrigation to incorporate into soil. Thus, zinc was not directly applied in maize cultivation season, residual effect of Zn applied in wheat was evaluated in current experiment. 

### 4.4. Plant Materials 

The experimental site was applied with herbicides glyphosate (1800 g ha^−1^ of a.i.) and 2,4-D (670 g ha^−1^ of a.i.) 15 days before plantation. Seeds were chemically treated with Standak Top®, a mix formulation of insecticide-imidacloprid + thiodicarb (45 g + 135 g of a.i. per 100 kg seeds) and fungicide-carbendazim + thiram (45 g + 105 g of a.i. per 100 kg seeds) a day before inoculations and plantation. A simple maize hybrid (FS500PWU-Forseed, registered with National Technical Commission on Biosafety of Brazil under reference no. 1596/2008 for tropical and sub-tropical regions) was sown on 18th November, 2019 and 12 November, 2020 in a no-tillage system at 3.3 seeds m^−1^. All treatments were applied with 350 kg ha^-1^ of NPK (08-28-16, urea) on the basis of soil analysis. Each experimental unit was 6 m long with 6 rows, 0.45 m apart with total plot size of 16.2 m^2^. Post-emergence herbicides atrazine + tembotrione (1000 + 84 g a.i. ha^−1^, respectively) were applied at V3 stage to control weeds. The topdressing fertilization of nitrogen (120 kg N ha^−1^ from ammonium sulphate) at V6 stage was performed in all treatments to uniformly distribute on soil surface. The crop was irrigated with pivot-irrigation system at 14 mm water volume according to the need of the crop. The crop was manually harvested on 2 March 2020 and 7 March 2021. 

### 4.5. Evaluations and Analysis

#### 4.5.1. Growth and Yield Attributes

Plant height was measured with meter-rod from ground to upper apex. Shoot dry matter was determined by harvesting four central lines, sun dried and weighed. Ten random ears were collected at harvest to count number of rows and grains ear^−1^plot^−1^. Hundred grains mass was measured with a precise scale on 13% humidity (wet basis). Ears were collected from central lines of each plot, threshed with electric thresher, and processed to calculate yield in kg ha^−1^ (productivity at 13% moisture content). The dried grains were then ground in a Willey mill for nutritional analysis.

#### 4.5.2. Nutritional Analysis

Twenty random leaves were collected from ear insertion at flowering stage in each plot. The plant material (shoot and grain) was collected at the time harvest. The samples were dried in an air-tight oven at 60 ± 5 °C for 72 h to attain uniform humidity. The material for each attribute was then individually grounded in a stainless-steel Willey knife mill by passing through a 10-mesh sieve in labeled plastic bags. Each sample was weighed (0.25 g), digested with nitroperchloric digestion (HNO_3_:HClO_4_ solution), and quantified by atomic absorption spectrophotometry following procedure of [23]. Zinc shoot and grains accumulation (g ha^−1^) were calculated from respective Zn concentration in shoot and grains, and dry matter yield ha^−1^, respectively. 

#### 4.5.3. Zinc Partitioning Index, Intake, and Use Efficiencies

Zinc partitioning index (ZPI) was calculated from the ratio of shoot Zn concentration to total (shoot + grains) Zn concentration in percent following Rengel and Graham [43]. Estimated Zn intake in Brazil (Equation (1)) was calculated from Zn biofortified grains of present study [44]. Brazil per capita maize consumption is around 24.69 kg person^−1^ year^−1^ (67.6 g person^−1^ day^−1^). Based on this information, estimated Zn intake of biofortified grains was calculated below in Equation (1).

Zn intake = [Zn grain] × C (1)

where Zn intake (g person^−1^ day^−1^) is daily estimated Zn consumption person^−1^, [Zn grain] (g kg^−1^) is Zn concentration in biofortified grains, and C (g person^−1^ day^−1^) is average maize consumption per person in Brazil [45]. 

Zinc use efficiency (ZnUE), agro-physiological efficiency (APE), recovery applied Zn (RAZn), and utilization efficiency (UE) were derived from the fractions of Zn concentration and accumulation in shoot and grains, dry matter, and grain yield following procedures of [22,46].

ZnUE = (GYF − GYWF) ÷ Zn applied dose(2)

APE = (GYF − GYWF) ÷ (ZnAF − ZnAWF) (3)

RAZn (%) = (ZnAF − ZnAWF) ÷ Zn applied dose(4)

UE = PE × RAZn (5)

where GYF = grain yield in Zn fertilized treatments, GYWF = grain yield in without Zn fertilized treatments, ZnAF = zinc accumulation in shoot + grain within fertilized treatments, ZnAWF = zinc accumulation in shoot + grain without fertilized treatments, and PE = physiological efficiency.

### 4.6. Statistical Analysis

The data were tested for normality with Shapiro–Wilk test which showed that data were normally distributed (W ≥ 0.90). The data were submitted to analysis of variance (F test). Zn soil application, and diazotrophic bacterial inoculations and their interactions were considered fixed effects in the model. When a main effect or interaction was observed significant by F test (*p* ≤ 0.05), then Tukey test (*p* ≤ 0.05) was used for comparison of means of residual soil Zn fertilization and diazotrophic bacterial inoculations [47]. 

The Pearson correlation analysis (*p* ≤ 0.05) was performed using R software [47]. To create a heatmap, the corrplot package was used, using the "color" and "cor.mtest" functions to calculate the coefficients and *p*-value matrices. 

## 5. Conclusions

Microbes-mediated Zn biofortification is one the most accessible, easy, and authentic strategies to increase Zn concentration and accumulation in the edible part of the maize crop. Our results indicated that residual Zn fertilization is a feasible and sustainable technique which has increased plant growth, yield, and Zn nutrition in both cropping seasons. The inoculation of diazotrophic bacteria along residual Zn fertilization performed better than without Zn fertilized treatments. Seed inoculation of *A. brasilense* and *B. subtilis* has increased height of insertion of first productive cob, plant height, shoot dry matter, and grain yield of maize under residual Zn fertilization. Most of the growth and yield attributes performed better with inoculation of *A. brasilense* in the absence of residual Zn fertilization. Zinc concentration in leaf, and accumulation in shoot and grains of maize were increased with *A. brasilense* and *P. fluorescens* under residual Zn fertilization. The highest Zn partitioning and daily Zn intake were also increased with inoculation of *P. fluorescens* and *A. brasilense* with residual Zn fertilization. All Zn efficiencies were increased with inoculation of *B. subtilis* except applied Zn recovery, which was greater with inoculation of *A. brasilense* when analyzed in residual Zn fertilized treatments. Therefore, inoculation of maize seeds with *B. subtilis* and *P. fluorescens* together with residual Zn fertilization could be an efficient alternative mechanism to improve Zn acquisition and use efficiencies, as well as productivity of maize in a sustainable manner in tropical savannahs. Prospective research aiming to improve Zn use efficiency and recovery with inoculation and co-inoculation of diazotrophic bacteria, and their influence on cereal biofortification, physiological, and molecular aspects is required to be carried out in different edaphic conditions to better understand Zn solubilizing bacteria under field conditions. 

## Figures and Tables

**Table 1:** First productive cob insertion, plant height, shoot dry matter, and grain yield of maize as influenced by diazotrophic bacteria and residual zinc doses in 2019–2020 and 2020–2021 cropping season.

Treatments	Plant Height		First Cob Insertion		Shoot Dry Matter		100 Grains Mass		Grain Yield	
	m				kg ha^−1^		g		kg ha^−1^	
	2019–2020	2020–2021	2019–2020	2020–2021	2019–2020	2020–2021	2019–2020	2020–2021	2019–2020	2020–2021
**Inoculations (I)**										
Without	2.66 ^b^	2.67 ^b^	1.22 ^b^	1.25 ^b^	11,945 ^b^	11,832	30.6 ^b^	31.7	7379	7307
*A. brasilense*	2.78 ^a^	2.79 ^a^	1.29 ^ab^	1.31 ^a^	12,642 ^a^	12,654	33.5 ^a^	34.4	8109	8233
*B. subtilis*	2.67 ^ab^	2.72 ^ab^	1.27 ^ab^	1.31 ^a^	12,381 ^a^	12,381	32.7 ^ab^	35.4	8449	8555
*P. fluorescens*	2.72 ^ab^	2.77 ^a^	1.29 ^a^	1.33 ^a^	12,355 ^a^	12,243	31.8 ^ab^	33.5	7911	7952
**Residual Zinc (Zn) Doses (kg ha^−1^)**										
0	2.67 ^b^	2.71 ^b^	1.25 ^a^	1.28 ^b^	12,102 ^b^	12,040	31.3 ^b^	32.7	7709	7806
8	2.72 ^a^	2.77 ^a^	1.28 ^a^	1.32 ^a^	12,559 ^a^	12,515	32.9 ^a^	34.8	8215	8218
**F-values**										
I	0.004 **	0.00 **	0.03 *	0.00 **	0.00 **	0.00 **	0.01 *	0.00 **	0.00 **	0.00 **
Zn	0.01 **	0.00 **	0.11^ns^	0.008 **	0.00 **	0.00 **	0.01 *	0.00 **	0.00 **	0.00 **
I × Zn	0.63 ^ns^	0.19 ^ns^	0.99 ^ns^	0.86 ^ns^	0.36 ^ns^	0.04 *	0.94 ^ns^	0.02 *	0.03 *	0.02 **
CV (%)	2.1	1.7	3.9	2.4	2.3	1.8	5.2	2.8	4.0	3.6

Means in the column followed by different letters are statistically different by Tukey test, *p* ≤ 0.05. ** and *—significant at *p* < 0.01 and *p* < 0.05, respectively, while ^ns^—non-significant by F-test.

**Table 2:** Leaf, shoot, and grain zinc (Zn) concentrations of maize as function of residual Zn doses and diazotrophic bacteria in 2019–2020 and 2020–2021 cropping season.

Treatments	Leaf Zn Concentration		Shoot Zn Concentration		Grain Zn Concentration	
	mg kg^−1^					
	2019–2020	2020–2021	2019–2020	2020–2021	2019–2020	2020–2021
**Inoculations (I)**						
Without (control)	20.6 ^b^	21.7	29.1 ^b^	29.5 ^b^	28.2 ^b^	32.5
*A. brasilense*	23.5 ^ab^	27.4	35.8 ^ab^	35.7 ^a^	35.4 ^a^	41.0
*B. subtilis*	23.9 ^ab^	28.0	33.4 ^ab^	33.9 ^ab^	32.9 ^a^	36.9
*P. fluorescens*	25.5 ^a^	29.1	37.7 ^a^	38.5 ^a^	34.6 ^a^	38.5
**Residual Zinc (Zn) Doses (kg ha** **^−1^** **)**						
0	21.7 ^b^	24.4	31.6 ^b^	31.9 ^b^	30.8 ^b^	34.1
8	25.0 ^a^	28.7	36.4 ^a^	36.9 ^a^	34.7 ^a^	40.3
**F-values**						
I	0.03 *	0.00 *	0.01 *	0.002 **	0.00 **	0.00 **
Zn	0.008 **	0.00 **	0.01 *	0.002 **	0.00 **	0.00 **
I × Zn	0.43 ^ns^	0.04 *	0.76 ^ns^	0.78 ^ns^	0.33 ^ns^	0.03 *
CV (%)	13.5	6.2	14.2	12.0	8.6	6.7

Means in the column followed by different letters are statistically different by Tukey test, *p* ≤ 0.05. ** and *—significant at *p* < 0.01 and *p* < 0.05, respectively, while ^ns^—non-significant by F-test.

**Table 3:** Shoot and grain zinc accumulation, zinc partitioning index, and estimated daily zinc intake by maize in Brazil as function of residual zinc fertilization and diazotrophic bacteria inoculations in 2019–2020 and 2020–2021 cropping seasons.

Treatments	Shoot Zn Accumulation		Grain Zn Accumulation		Zn Partitioning Index		Zn Intake (Brazil)	
	g ha^−1^				%		g person^−1^ day^−1^	
	2019–2020	2020–2021	2019–2020	2020–2021	2019–2020	2020–2021	2019–2020	2020–2021
**Inoculations (I)**								
Without I	348.5 ^b^	349.3 ^b^	208.9	237.7	51.8 ^a^	47.5 ^a^	1.9 ^b^	2.2
*A. brasilense*	452.8 ^a^	453.4 ^a^	286.7	337.6	50.2 ^a^	46.6 ^a^	2.4 ^a^	2.7
*B. subtilis*	413.5 ^ab^	420.0 ^ab^	279.5	317.9	50.1 ^a^	47.8 ^a^	2.2 ^a^	2.5
*P. fluorescens*	466.3 ^a^	471.6 ^a^	273.9	306.2	51.8 ^a^	49.8 ^a^	2.3 ^a^	2.6
**Residual Zinc (Zn) Doses (kg ha^−1^)**								
0	383.4 ^b^	384.2 ^b^	238.2	266.9	50.5 ^a^	48.1 ^a^	2.1 ^b^	2.3
8	457.2 ^a^	463.0 ^a^	286.3	332.7	51.1 ^a^	47.8 ^a^	2.4 ^a^	2.7
**F-values**								
I	0.002 **	0.00 **	0.00 **	0.00 **	0.86 ^ns^	0.31 ^ns^	0.00 **	0.00 **
Zn	0.001 **	0.00 **	0.00 **	0.00 **	0.71 ^ns^	0.77 ^ns^	0.00 **	0.00 **
I × Zn	0.63 ^ns^	0.80 ^ns^	0.04 *	0.00 **	0.99 ^ns^	0.78 ^ns^	0.33 ^ns^	0.02 **
CV (%)	13.7	12.5	8.9	7.3	8.6	6.8	8.6	6.6

Means in the column followed by different letters are statistically different by Tukey test, *p* ≤ 0.05. ** and *—significant at *p* < 0.01 and *p* < 0.05, respectively, while ns—non-significant by F-test.

**Table 4:** Zinc efficiencies of maize as function of residual zinc fertilization and diazotrophic bacteria inoculations in 2019–2020 and 2020–2021 cropping seasons.

Treatments	ZnUE		APE		UE		AZnR	
	kg kg^−1^						%	
	2019–2020	2020–2021	2019–2020	2020–2021	2019–2020	2020–2021	2019–2020	2020–2021
**Inoculations (I)**								
Without (control)	164 ^c^	68 ^c^	19 ^a^	4.5 ^b^	297 ^b^	131 ^b^	12 ^b^	15 ^b^
*A. brasilense*	233 ^b^	178 ^b^	7 ^a^	3.8 ^b^	483 ^a^	353 ^a^	35 ^a^	47 ^a^
*B. subtilis*	317 ^a^	270 ^a^	10 ^a^	6.5 ^a^	509 ^a^	381 ^a^	32 ^a^	42 ^a^
*P. fluorescens*	190 ^c^	135 ^b^	7 ^a^	3.2 ^b^	379 ^b^	219 ^b^	33 ^a^	43 ^a^
**F-values**								
I	0.00 **	0.00 **	0.05 *	0.003 **	0.00 **	0.00 **	0.008 **	0.00 **
CV (%)	8.3	13	54	20	10	17	29	21

ZnUE = Zinc use efficiency, APE = Agro-physiological efficiency, UE = Utilization efficiency, and AZnR = Applied zinc recovery. Means in the column followed by different letters are statistically different by Tukey test, *p* ≤ 0.05. ** and *—significant at *p* < 0.01 and *p* < 0.05, respectively, while ^ns^—non-significant by F-test.

**Table 5:** Pre-maize experiments soil analysis of composite sample in a soil layer (0–0.20 m) in 2019–2020 and 2020–2021 cropping seasons.

Properties	Units	Status	
		2019–2020	2020–2021
pH (CaCl_2_)	----	5.2	5.3
Organic matter	mg dm^−3^	18	23
P (resin)	mg dm^−3^	38	40
K	mmol_c_ dm^−3^	1.7	1.9
Ca	mmol_c_ dm^−3^	21	22
Mg	mmol_c_ dm^−3^	15	12
B (hot water)	mg dm^−3^	0.14	0.39
Cu (DTPA) *	mg dm^−3^	3.4	3.7
Fe (DTPA) *	mg dm^−3^	25	28
Mn (DTPA) *	mg dm^−3^	38.1	37.3
S-SO_4_	mg dm^−3^	4.0	22
H + Al	mmol_c_ dm^−3^	34	31
CEC (pH7) *	mmol_c_ dm^−3^	75.7	66.9
V *	%	50	54
Zn content (DTPA)			
Without Zn fertilization	mg dm^−3^	0.9	1.1
Residual Zn fertilization	mg dm^−3^	2.2	3.0

* CEC: cation exchange capacity, V: base saturation, DTPA: Diethylenetriaminepentaacetic acid.

## Data Availability

Not applicable.
